# An EEG-based asynchronous MI-BCI system to reduce false positives with a small number of channels for neurorehabilitation: A pilot study

**DOI:** 10.3389/fnbot.2022.971547

**Published:** 2022-09-12

**Authors:** Minsu Song, Hojun Jeong, Jongbum Kim, Sung-Ho Jang, Jonghyun Kim

**Affiliations:** ^1^Department of Medical Device, Korea Institute of Machinery and Materials, Daegu, South Korea; ^2^School of Mechanical Engineering, Sungkyunkwan University, Gyeonggi-do, South Korea; ^3^Department of Robotics Engineering, Daegu Gyeongbuk Institute of Science and Technology, Daegu, South Korea; ^4^Department of Physical Medicine and Rehabilitation, College of Medicine, Yeungnam University, Daegu, South Korea

**Keywords:** brain-computer interface, brain plasticity, contamination, false positive rejection, motor imagery, neurorehabilitation

## Abstract

Many studies have used motor imagery-based brain–computer interface (MI-BCI) systems for stroke rehabilitation to induce brain plasticity. However, they mainly focused on detecting motor imagery but did not consider the effect of false positive (FP) detection. The FP could be a threat to patients with stroke as it can induce wrong-directed brain plasticity that would result in adverse effects. In this study, we proposed a rehabilitative MI-BCI system that focuses on rejecting the FP. To this end, we first identified numerous electroencephalogram (EEG) signals as the causes of the FP, and based on the characteristics of the signals, we designed a novel two-phase classifier using a small number of EEG channels, including the source of the FP. Through experiments with eight healthy participants and nine patients with stroke, our proposed MI-BCI system showed 71.76% selectivity and 13.70% FP rate by using only four EEG channels in the patient group with stroke. Moreover, our system can compensate for day-to-day variations for prolonged session intervals by recalibration. The results suggest that our proposed system, a practical approach for the clinical setting, could improve the therapeutic effect of MI-BCI by reducing the adverse effect of the FP.

## Introduction

Brain–computer interface (BCI) using electroencephalogram (EEG) signals is gaining significance in stroke neurorehabilitation owing to its positive effect on rehabilitation (Friehs et al., 2004; Lebedev and Nicolelis, 2006; Daly and Wolpaw, 2008; Grosse-Wentrup et al., 2011; Young and Tolentino, 2011; Bai et al., 2020). Rehabilitative BCI systems use EEG signals to provide motor-related neurofeedback immediately after the motor intention to generate a planning execution cycle. By repeating this cycle, brain plasticity can be induced by firing mirror neurons to reorganize the damaged neural circuits in the brain (Bennett et al., 1964; Livingston, 1966; Murphy and Corbett, 2009; Duffau, 2016; Reinkensmeyer et al., 2016; Sasmita et al., 2018). Many studies have shown that these BCI systems can improve the rehabilitation results in patients with stroke by increasing motor function after the training sessions (Friehs et al., 2004; Lebedev and Nicolelis, 2006; Daly and Wolpaw, 2008; Murphy and Corbett, 2009; Grosse-Wentrup et al., 2011; Young and Tolentino, 2011; Bai et al., 2020).

Rehabilitative BCI systems can be classified into two types: synchronous and asynchronous. The synchronous system, which detects target brain signals during a pre-defined time after a visual or sound cue is provided to the users (Pfurtscheller et al., 2003), is inappropriate for training protocols based on the activities of daily living (ADL) as users cannot freely control BCI whenever desired. In contrast, the asynchronous system, which keeps monitoring until the target brain signal is detected (Leeb et al., 2007; Diez et al., 2011; Chae et al., 2012; Kus et al., 2012), is beneficial to rehabilitative BCI as it can provide a more ADL-like experience (Aricò et al., 2020). Since the asynchronous system monitors the brain signal continuously, the feedback of the system can be provided not only in the user-intended time (true positive; TP) but also for the rest of the time (false positive; FP).

For the asynchronous system of rehabilitative BCI, event-related desynchronization (ERD), an attenuating power on certain frequency (alpha and beta) bands, is a typical feature (Pfurtscheller and Lopes Da Silva, 1999). Motor execution (ME) results in ERD; however, most patients with stroke have difficulties performing ME due to motor impairments. Hence, ERD caused by motor imagery (MI) has been regarded as an alternative to ME ERD. This is supported by the following facts. MI ERD shares almost the same activation area and frequency band if the participant performs the exact image of the desired motor task (Miller et al., 2010; Jeong et al., 2019), and motor function recovery after MI training has been reported in patients with stroke (Sun et al., 2016).

It is well known that asynchronous BCI systems are more complicated than their synchronous counterparts (Nicolas-Alonso and Gomez-Gil, 2012; Hramov et al., 2021). Moreover, MI is a complex mental task, namely, intention, tactile, proprioceptive, and visual feeling of the specific motor task (Jeannerod, 2006); thus, MI ERD generated by stroke patients with chronic motor impairments would be weak, leading the asynchronous system to become more challenging. To solve this challenge, a study used an additional electromyogram (EMG) sensor to deliver synchronous-like situations in an asynchronous system (Bhagat et al., 2016); however, this scheme can only be used by a minority of patients with stroke who can provide sufficient EMG on the limb. In contrast, many studies have used spatial pattern-based detection methods, such as spatially applied linear discriminant analysis (Lew et al., 2012; Mrachacz-Kersting et al., 2017), independent component analysis (Ahmadian et al., 2013), and common spatial patterns (Wang et al., 2005; Blankertz et al., 2008), to increase the overall accuracy of BCI based on MI (Hortal et al., 2015; Mrachacz-Kersting et al., 2017); thus, these methods could be applied to overcome the aforementioned challenges. However, it is inappropriate for use in rehabilitative BCI in the clinical environment as they require excessive EEG channels, which leads to a lack of motivation and a decrease in concentration due to fatigue. Note that there were a few attempts to reduce the number of channels; however, they were not successful owing to the significant deterioration of their accuracy (Arvaneh et al., 2011; Tam et al., 2011).

To provide rehabilitative BCI to a broad patient population and to reduce the number of EEG channels, single-channel-based MI detection, also called as brain-switch has been attempted (Müller-Putz et al., 2010; Ge et al., 2014; Camacho and Manian, 2016; Chen et al., 2017; Ko et al., 2017). However, some previous studies just focused on the increase of TP detection and it leads to an increase in FP detection (Camacho and Manian, 2016). The increased number of FPs is more dangerous than decreasing TP of MI from a rehabilitation perspective as the wrong-directed neural cycle could induce inappropriate (pathologic) brain plasticity and interfere with the improvement of MI skills (Barbero and Grosse-Wentrup, 2010; Grosse-Wentrup et al., 2011; Liu et al., 2013; Alimardani et al., 2014; Niazi et al., 2022).

An alternate approach to reduce FPs in ERD is to identify possible sources of the signals that can be confused. The possible sources can be considered non-region of interest (non-ROI) channels for MI task, whereas the region of interest (ROI) channels are interesting channels for investing the effects of the MI task, which is generally contralateral motor area (Kober et al., 2019). Some brain signals (other movement-related signals and cognitive task signals) and the EMG signals generated by eye movement, contraction of the frontalis, temporalis, and neck muscles can be formed as alpha and beta attenuation, similar to MI alpha and beta rhythm (Goncharova et al., 2003). They can be reduced by experimental instructions or easily rejected by using EOG/EMG sensors. The sensory-related signals such as visual evoked potential (VEP) and auditory evoked potential (AEP) also show ERD-like short-lasting attenuation in the alpha and beta bands in non-motor areas (Makeig, 1993; Salenius et al., 1995; Duarte et al., 2009; Toscani et al., 2010; Oppitz et al., 2015). However, the visual/auditory stimuli are generally used in BCI but are difficult to eliminate with external sensors or experimental instructions. Especially, patients with stroke lack attention and require various types of visual and auditory aids to properly concentrate on the rehabilitative BCI (Thaut and McIntosh, 2014; Loetscher and Lincoln, 2019). Therefore, identifying and rejecting these signals could minimize the expected FPs during BCI sessions.

This study addressed a detection and rejection algorithm for a fully asynchronous BCI system using MI ERD. We first identified the sensory-related signals, which could confound individual MI ERD. Then, based on the characteristics of the signals, we designed a classifier for an asynchronous BCI system to detect MI ERD and reject FPs by combining (1) a single-channel-based MI detection in ROI and (2) a non-region of interest (non-ROI) channel-based FP rejection algorithm originating from our previous work (Song et al., 2018). Through experiments with healthy participants and patients with stroke, the validity of the idea of a non-ROI channel was investigated, and the MI detection performance of the proposed classifier was evaluated using both offline simulations and online BCI sessions.

## Methods

### Classifier design for rejecting EEG contamination

The experimental protocol for MI-based BCI generally contains a calibration session before the BCI. In this study, the calibration session not only extracts training data but also screens ROI and non-ROI candidate channels. Note that, in this study, ROI indicates an EEG channel that contains the origin of our interested EEG signal (i.e., MI) and non-ROI indicates the region made of EEG channels outside of our interested area, where we define the sources that EEG contaminations occur. Along with the MI task, the session included several paradigms for screening the source of EEG contamination from sensory (visual/auditory) stimuli: (1) VEP from action observation, (2) VEP from non–motor-related various themed images, and (3) AEP from the auditory cue. The VEP from action observation represents passive action observation in a rehabilitation environment and action recollection that may occur during rest, which refers to unintended cognitive activity that unconsciously reminds the patient of exercise execution. For another VEP, the various themed images were intended to induce unwanted non–motor-related cognition tasks by showing different images for each trial, to mimic the lack of concentration of patients with stroke. The AEP represents miscellaneous auditory cues and sound originated diversions in the rehabilitation environment, which attract attention from the patient. VEPs are known to have a negative peak in the alpha band in the posterior–occipital area (Salenius et al., 1995; Toscani et al., 2010), and the AEP is known to show negative oscillations in the alpha band in the temporal and midline areas (Makeig, 1993; Duarte et al., 2009; Oppitz et al., 2015). Considering these characteristics, we designed paradigms to reveal the time-frequency patterns of EEG contamination that can be used to develop classifiers for rejecting them.

#### Selection of ROI and non-ROI EEG channels

Instead of applying conventional spatial filters with many EEG channels to overcome the limitation of weak MI ERD, we used a small number of channels and algorithm following characteristic of EEG signals: the EEG signals radially flow through the scalp-like electrocortical ripple, affecting nearby electrodes (Salenius et al., 1995; Mcfarland et al., 1997). If MI ERD appears in the EEG channel located in the MI-related area (ROI channel), we could find similarly desynchronized power of signals on the nearby channels; however, the signals would be weaker than the ROI channel due to skin impedance. In contrast, if stronger power desynchronization appeared in the channel located outside of the MI-related area (non-ROI channel) when ERD is detected in the ROI channel, the detected power desynchronization can be regarded as pseudo-MI ERD, originating from the non-ROI channel due to EEG contamination. This means that the use of proper non-ROI channel information could enable the effective discrimination of pseudo-MI ERD without using many EEG channels.

**Figure 1:**
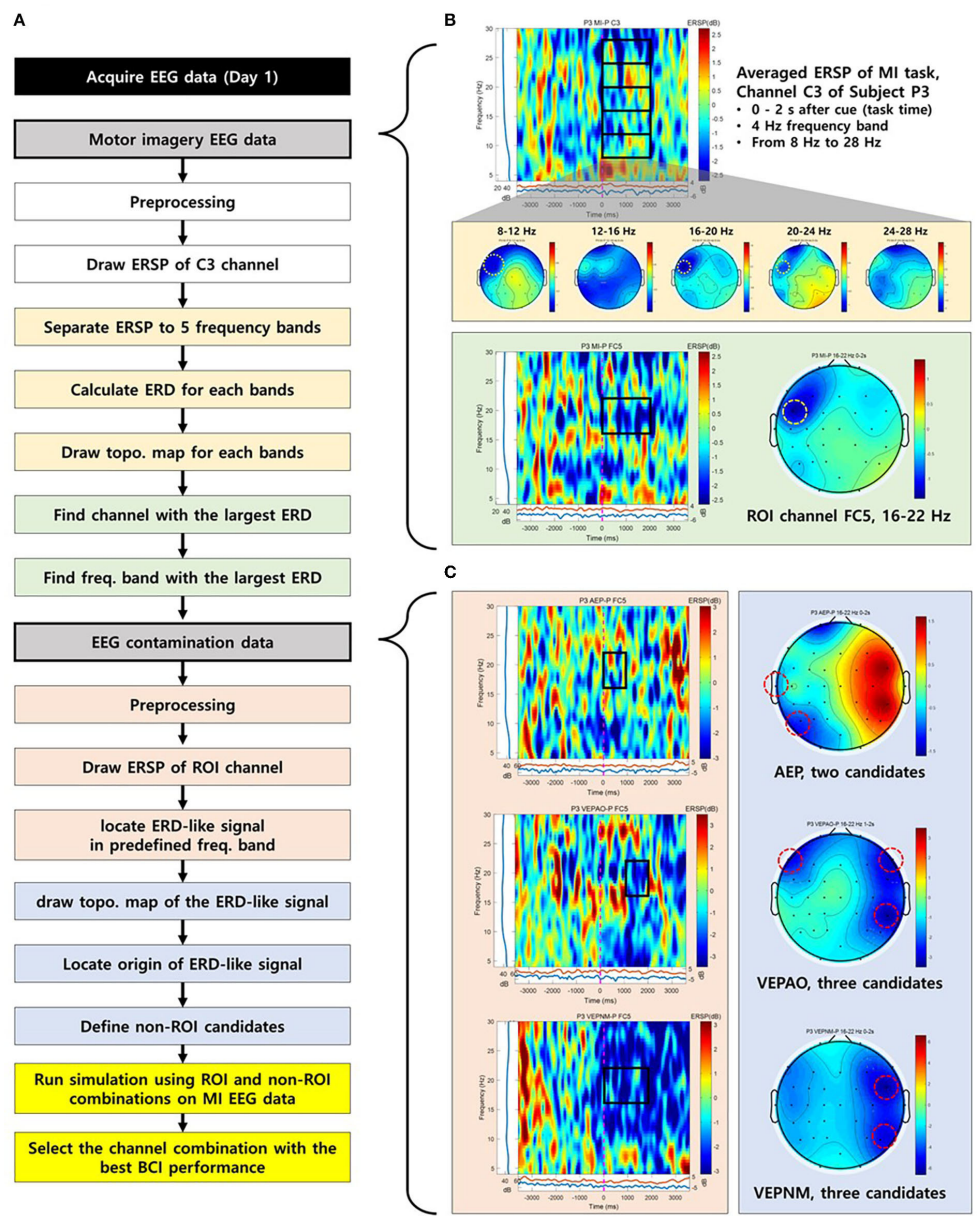
The process to select a region of interest (ROI) and non-ROI candidate channels of a representative participant (P3). **(A)** The flow chart for the process to select ROI and non-ROI candidate channels. **(B)** The process to select a ROI. The yellow dotted circle on the topographical map represents the selected ROI. **(C)** Non-ROI candidate channels. The red dotted circle on the topographical map represents the source of electroencephalogram (EEG) contamination and candidate channels for non-ROI.

To implement the approach above, ROI and non-ROI channels for individuals should be carefully selected. Figure 1A is a diagram of a workflow of channel selection, which is colored by a group of tasks that can be represented by characteristic example figures (Figures 1B,C). Figure 1B shows a characteristic example of the selection process of the ROI channel. Based on the event-related spectral perturbation (ERSP) map of the MI task, we extracted the data during the MI task in five frequency bands from 8 Hz to 28 Hz (mu and beta) with 4 Hz intervals (yellow boxes in Figures 1A,B). Note that EEGLAB functions were used to calculate ERSP and sinusoidal wavelet (short-time DFT) transform was used for the computation of spectral estimate (Delorme and Makeig, 2004). Baseline correction is applied to ERSP based on the pre-stimulus segment (−4 to −2 s from the cue). Then, the averaged ERSP in the task period for each band data was drawn into a topographical map (Figure 1B), and a channel that was closest to the area, where it showed the lowest averaged ERSP power among all five maps, was chosen as the ROI channel, the source channel of MI ERD (yellow dotted circle on the topographical map in Figure 1B). Finally, we drew the ERSP map of the chosen channel to specify the frequency band of the MI ERD (green box in Figure 1B). The ROI channel selection was double-checked by drawing the topographical map of the frequency band specified to see whether the selected channel showed the strongest ERD (Figure 1B).

The candidates for the non-ROI channel were also chosen as follows: To find the source channel of EEG contamination, the inducers of contamination, visual and auditory stimulation, were provided to the participants. Based on the determined frequency band in ROI selection, the ERSP map and topographical maps of the ROI for the paradigms were drawn. Then, the sources showing the lowest averaged ERSP values in the topographical maps were chosen as candidates for the non-ROI channel (Figure 1C). Offline MI-BCI simulation was performed in a proposed classifier with multiple combinations of candidates to determine the best combination with the highest FP rejection rate as a non-ROI channel.

#### Classifier structure

**Figure 2:**
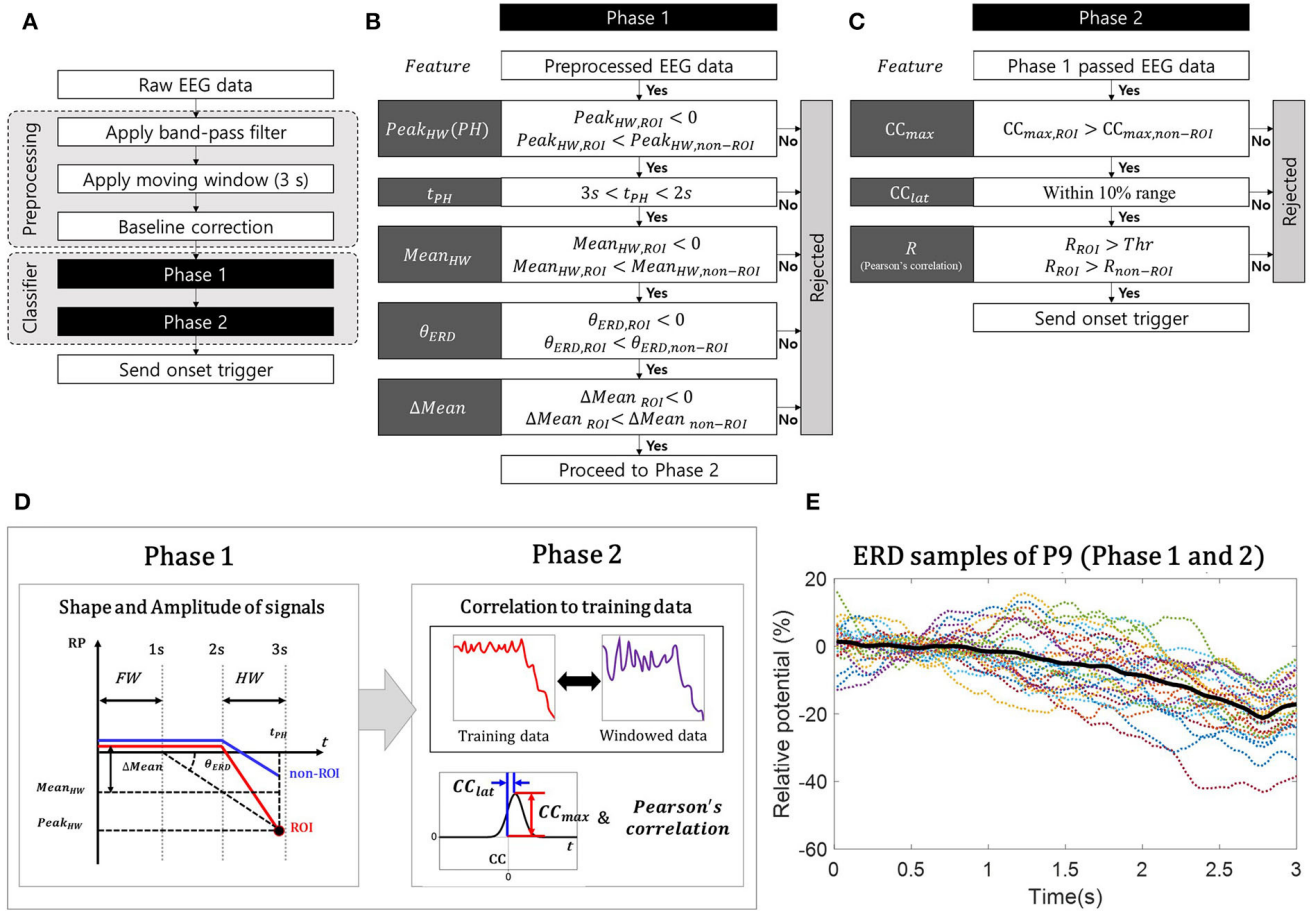
Simple illustrations of two-phase classifier algorithm. **(A)** Flow chart for the whole algorithm; **(B)** Flow chart for phase 1: an illustration of the event-related desynchronization (ERD)-like shape of the band power relative potential (RP) signal and its features; **(C)** Flow chart for phase 2: cross-correlation coefficients (*CC*), latency (*CC*_*lat*_), and Pearson's correlation, R; **(D)** Structure of proposing classifier; and **(E)** Characteristic of ERD samples used in classifier training (participant P9).

The classifier, which contains the detection algorithm of MI ERD and rejection algorithm of pseudo-MI ERD, was designed with the following hypothesis: The ERD signals generated by EEG contamination spread-like radial waves from the non-ROI channel and affect the ROI channel as pseudo-MI ERD. Based on this hypothesis, the proposed classifier was built by comparing the non-ROI channels obtained from various EEG contamination paradigms with the ROI channels obtained from the MI paradigm. It should be noted that we validated this hypothesis using experimental data in this study. To detect the desired feature of MI ERD by rejecting pseudo-MI ERD, the proposed classifier adopted a two-phase structure, MI-ERD detection method in a single ROI channel, and FP rejection method with non-ROI channels as illustrated in Figure 2A.

We first specified the characteristics of the 3-s-long windowed signal that we wanted to detect as MI ERD. For the feature of the proposed classifier, the relative potential (RP) was used for the ERD calculation method (Pfurtscheller and Lopes Da Silva, 1999). The length of the windowed signal was 3 s, to distinguish the feature by including the signal during “rest” before the ERD begins. The desired characteristic of the signal was basically to contain the negative peak of ERD; however, we also considered that the peak is located at the hind area of the windowed signal, as illustrated in Figure 2D, to minimize its detection latency (Song et al., 2018). Based on this characteristic, the first phase was to distinguish the promising MI ERD from the incoming windowed signals using the features for describing the shape and amplitude of the signal. After dividing the signal into the front and hind areas (Figure 2D), we calculated the following features: the minimum peak in the hind area (*Peak*_*HW*_) and its timing (*t*_*PH*_), mean band power value of the hind area (*Mean*_*HW*_)), mean band power difference between the areas (*Mean* = *Mean*_*FW*_−*Mean*_*HW*_), and decline angle of ERD (θ_*ERD*_), as summarized in Figure 2B. The decline angle was calculated as follows:


(1) $$\begin{array}{l} \theta_{ERD}\;=\;\mathrm{atan}\left(\frac{Peak_{HW}-Mean_{FW}}{t_{PH}-t_{FW}}\right) \end{array}$$

where *t*_*FW*_ denotes the timing of the baseline, determined as the end of the front area. Using the features of the ROI and non-ROI channels, we constructed the conditions to find the promising MI ERD, as shown in Figures 2B,C, based on the following statement originating from the hypothesis: the signal on the ROI channel would be MI ERD when it has the lowest peak (*Peak*_*HW,ROI*_ < 0) and average amplitude (*Mean*_*HW*_) in the hind window (3*s*< *t*_*PH*_ < 2*s*), with larger amplitude (*Peak*_*HW,ROI*_ < *Peak*_*HW,non*−*ROI*_, and *Mean*_*HW,ROI*_ < *Mean*_*HW,non*−*ROI*_), and larger reduced amplitude (*Mean* = *Mean*_*FW*_−*Mean*_*HW*_) and deeper decline angle (θ_*ERD*_ < 0) compared with pseudo-MI ERD on the non-ROI channels (*Mean*_*ROI*_ < *Mean*_*non*−*ROI*_, and θ_*ERD,ROI*_ < θ_*ERD,non*−*ROI*_).

In the second phase, the following correlation-related features between each windowed signal (on the ROI and non-ROI channels) and the actual MI ERD signal collected as training data were calculated: the maximum value of the cross-correlation coefficients (*CCmax*), latencies of the coefficients (*CClat*) (Lewis, 1995; Sadeghian and Moradi, 2008; Chandaka et al., 2009; Siuly and Li, 2012), and normalized Pearson's correlation coefficients (*R*) (Pearson, 1895). The similarity between the training data and windowed signal on the ROI was evaluated using *CClat* and *R* (Figure 2C). Moreover, by comparing *CCmax* and *R* from the ROI with those from the non-ROI, we checked whether the windowed signal on the ROI was more similar to the training data than the windowed signal on the non-ROI (Figure 2C). Note that the correlation features, *CCmax* and *R*, are insensitive to the magnitude of the signals. Since our algorithm relies on a relative comparison between ROI and non-ROIs, we focused on detecting the similarity with training data from calibration session rather than its magnitude.

### Participants and experimental design

The experiment comprised two sessions. In the calibration session, we measured the participant's EEG behavior when performing a targeted MI task and when exposed to different sensory stimulations. After a few days, the MI-BCI session was conducted based on the classifier that was calibrated for each participant using the data obtained from the calibration session, to evaluate the performance of the proposed MI-BCI system.

Eight healthy young adults (four men, four women, average age: 22.8 ± 4.1 years) and nine patients with stroke (seven men, two women, average age: 56.7 ± 7.9 years) who were in the chronic stage post-stroke for 124.2 ± 42.7 months were recruited in this study. All healthy participants were right-handed with no history of brain–nervous system injuries or neurological diseases. Seven patients had a hemorrhage in the left hemisphere, resulting in hemiplegia on the right upper limb, while the other two patients had the opposite. Note that three of the eight healthy participants did not participate in the MI-BCI session owing to personal reasons. With the approval of the institutional review board (DGIST-170721-HR-025-08), all participants voluntarily signed their consent after the experimental details were provided.

**Figure 3:**
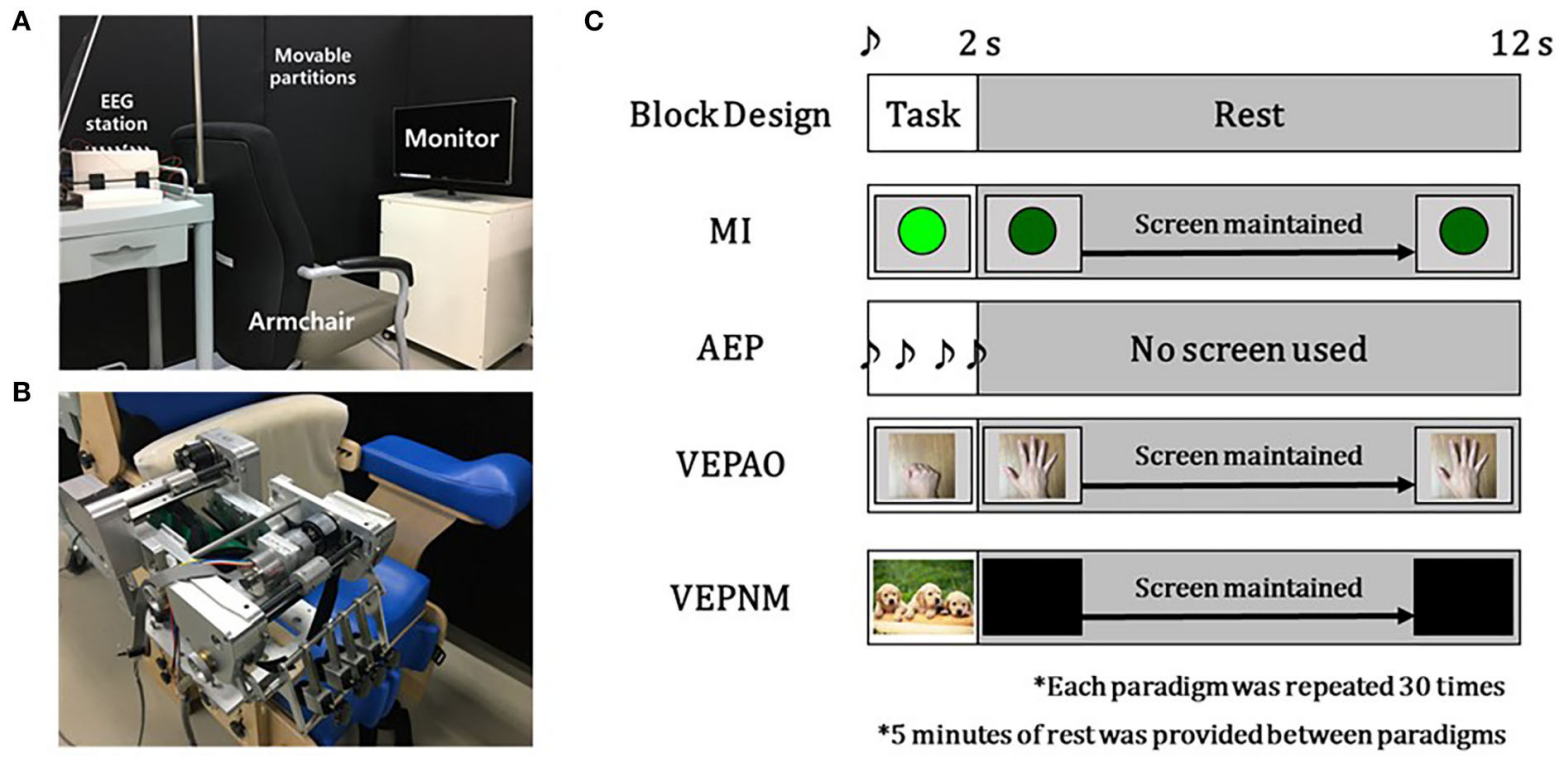
Experiment setup and block design of the Paradigms. **(A)** Experiment setup for the measurement session. **(B)** Exoskeleton hand rehabilitation robot setup for the motor imagery-based brain–computer interface (MI-BCI) session. **(C)** Block design-based experiment paradigms. (VEPAO: VEP with action observation; VEPNM: non–motor-related VEP).

Figure 3 shows the experimental setup. The experiment was performed in a quiet and air-conditioned room with minimal visual artifacts blocked by partitions (Figure 3A). A custom hand exoskeleton robot was used to provide the participant's fist open/close motor feedback for the MI-BCI session (Bae et al., 2017; Lee et al., 2017) (Figure 3B). The classifier for the MI-BCI session was implemented using customized OpenVibe (Inria, France), Python, and LabVIEW (National Instruments, USA) codes.

**Figure 4:**
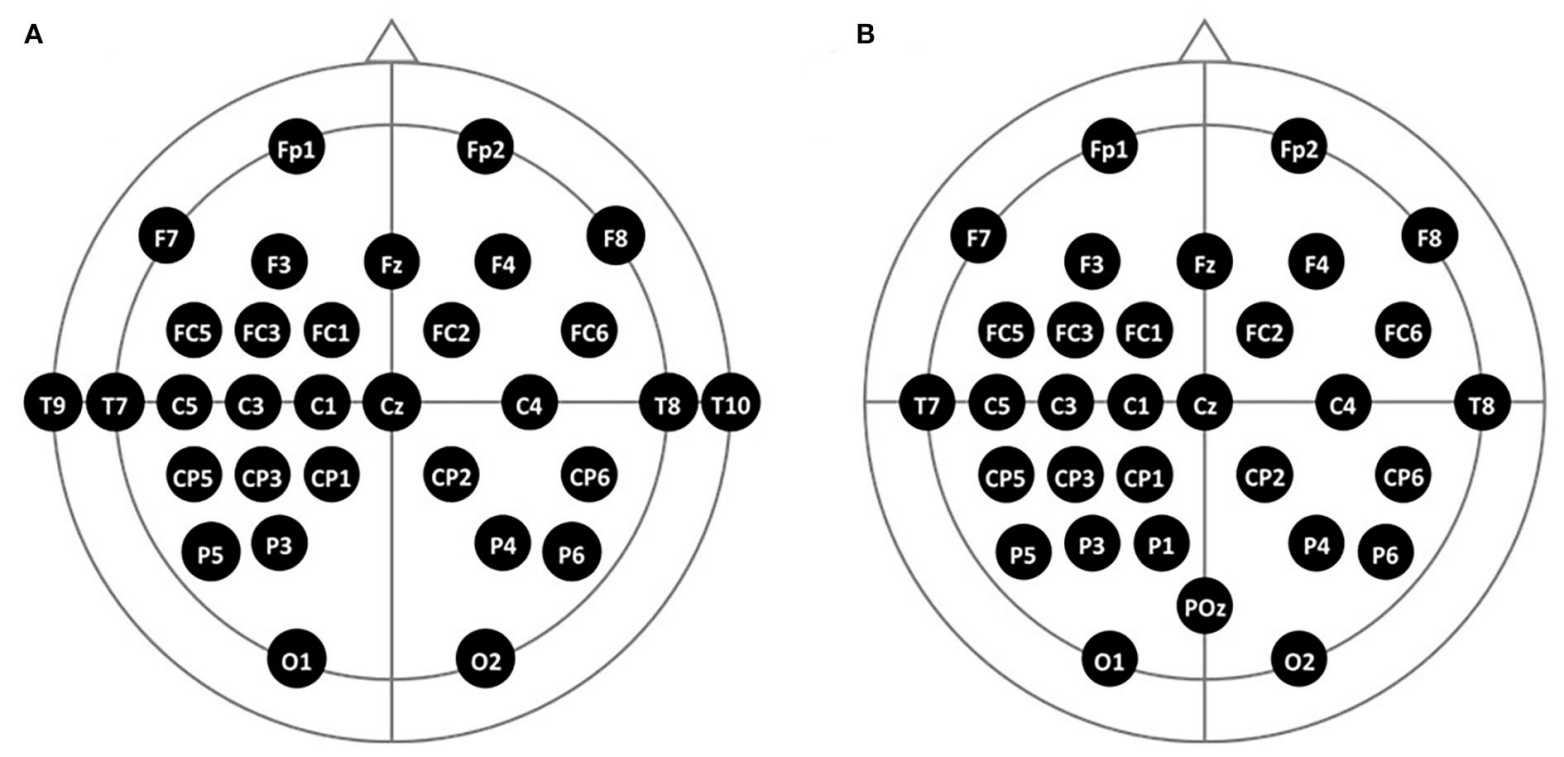
The 10–20 system-based 32-channel locations of two participant groups. **(A)** Locations of the healthy group and **(B)** locations of the stroke group.

We used a 32-channel EEG (Active Two EEG, BioSemi Co. Ltd., Netherlands), in which electrodes were attached to a 64-channel EEG cap (FLASH type EEG holder, Shimadzu Corp., Japan) based on a 10–20 system. The channel locations were widespread and densely distributed on the left motor cortex to locate the ROI channel when the participants imagined right-handed movement, as illustrated in Figure 4. For better convenience for the patient group, two channels on each temporal area (T9 and T10) were relocated to the left and medial parietal areas (P1, POz) (Figure 4).

### Protocols

#### Calibration session

The participant sat on a chair with an armrest in front of the monitor leveled on the eye level (Figure 3A). Each paradigm shared the same block design as that described in Figure 3C. During the rest period, the other cognitive actions were restricted. The single beep sound was played for 0.25 s at the beginning of the task period to notify the participants. The task period of each paradigm had its event cue and certain cognitive or motor tasks for 2 s (Figure 3C). In the MI paradigm, the participants were asked to perform pure MI of opening and closing the fist for a single time after a visual cue (green circle) with closing their fist of the right hand (or affected hand for the patient group) during the task period (Figure 3C).

For the paradigm to measure AEP, the participant was asked to relax and to concentrate on the sound cue (a beep sound repeated four times at 0.5 s intervals) provided during the task period (Figure 3C). For VEP, we measured two types of VEP: VEP with action observation and non–motor-related VEP. In both paradigms for the VEPs, the participants were asked to relax without any movement and to watch the monitor. In each VEP paradigm, the monitor displayed a top-view image of the fist open/close for the former VEP and random images not related to the motor task for the latter VEP (Figure 3C), which turned black during the rest period for proper relaxation.

During the calibration session, the participants participated in paradigms in the following order: MI was performed first to avoid the influence of other EEG contamination paradigms, and following AEP, VEP with action observation, and non–motor-related VEP were performed in randomized order. The block for each paradigm was repeated 30 times for 7 min, with 5 min of rest between each paradigm.

#### MI-BCI session

First, the participants were asked to perform the MI paradigm of the calibration session as practice, as the last MI was performed a few days ago. During the paradigm, the operator monitored the classifier and slightly adjusted the threshold for Pearson's product-moment correlation coefficient (PPMCC) from the second phase of the classifier to compensate for the day-to-day variation. After the MI paradigm, the hand exoskeleton robot was attached to the chair armrest, the right side for healthy participants and affected side for patients with stroke (Figure 3B), and the participants were put on the robot for the MI-BCI session.

**Figure 5:**
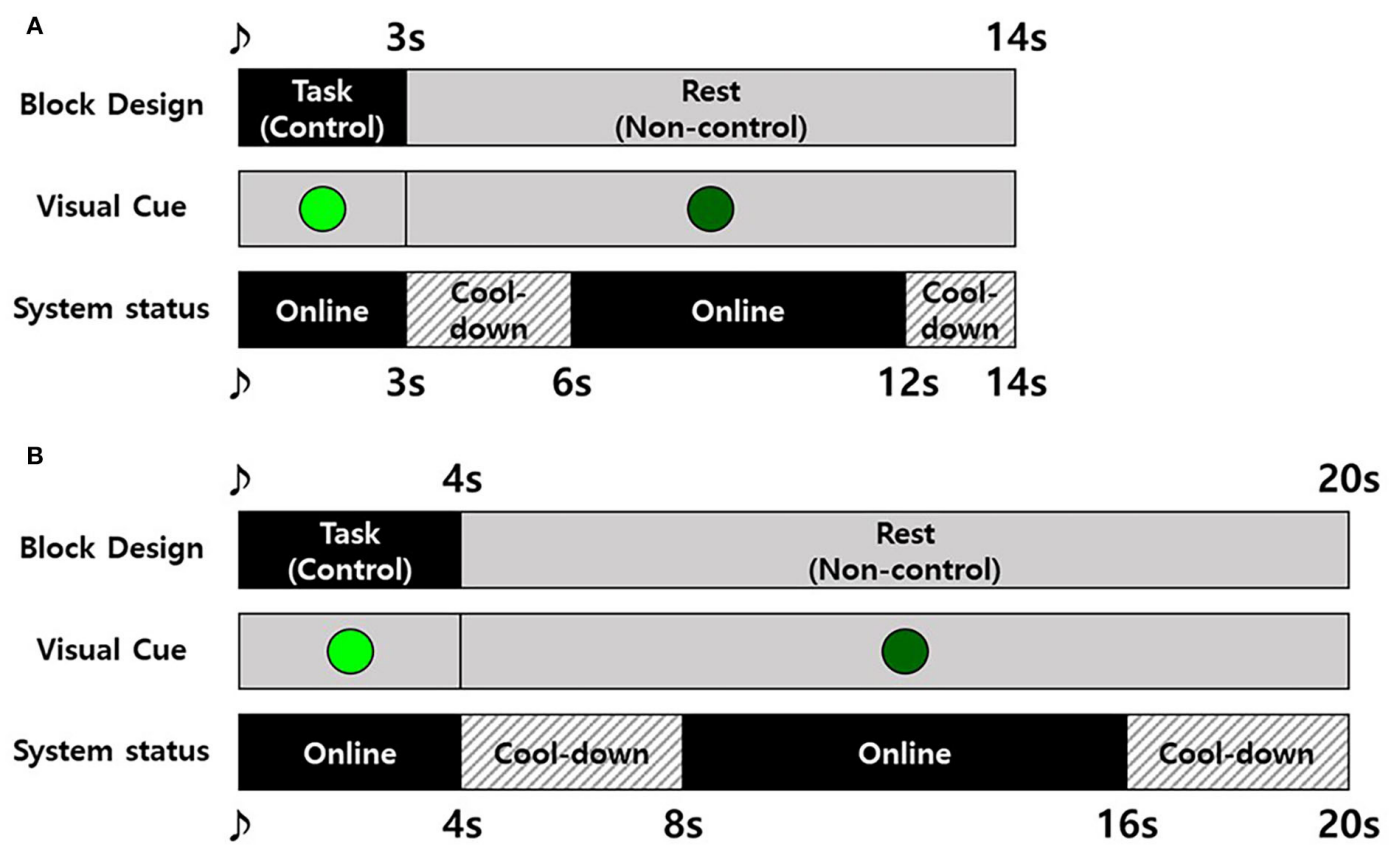
Experiment block design for the motor imagery-based brain–computer interface (MI-BCI) session. **(A)** The healthy participant group and **(B)** stroke participant group.

In the MI-BCI session, participants were instructed to perform MI to open and close their fist for a single time during the task (control) period and asked to remain as calm as possible in the rest (non-control) period (Leeb et al., 2007) following block design, as shown in Figure 5. Here, we used a synchronous block design for evaluating asynchronous MI-BCI systems as there are no observable signs to confirm the execution of MI. The BCI system went to the offline state (cool-down) immediately before and after the task period (Figure 5). Except for the cool-down status, the system was online to wait for the detection of MI, and the detection resulted in movement feedback by the robot. The cool-down was used to calm the brain signals after MI and/or movement feedback. If the detection occurred during the task period, it was considered TP, and if it occurred during the rest period, it was considered FP. The number of TPs and that of FPs were counted to evaluate the performance of the proposed MI-BCI system.

The experimental block designs for the healthy and patient groups were slightly different (Figure 5) because the patients with stroke felt more difficulty performing MI and took more time to concentrate than healthy participants. To compensate for the burden increase due to a longer task period, the time of rest period and cool-down were also increased to maintain the time ratio between control, non-control, and cool-down.

### Data analysis

#### Data processing

To investigate the source of EEG contamination, raw EEG data from the calibration session were epoched based on the time information of the cue (−4 to 4 s from the cue). Epoched data were normalized by subtracting the baseline, which is the averaged data from −4 to −2 s based on the cue. A baseline-corrected data epoch was used to plot the time-frequency information map and ERSP map. As mentioned in Method.1.a, five frequency bands were extracted from the ERSP map and used to draw topographical maps to select the participant-specific frequency band, ROI channel, and candidates of the non-ROI channel.

To validate our hypothesis for the proposed classifier, RP was calculated the same as the ERD calculation for the feature of the proposed classifier as follows:


(2) $$\begin{array}{l} \left(A-R\right)/R\times 100 \end{array}$$

where *A* is the power of filtered data and *R* is the power of preceding baseline data (Pfurtscheller and Lopes Da Silva, 1999). To calculate the RP, 8 s data epochs (−4 to 4 s from the cue) were extracted based on the time information in the filtered data, and the band power of the epoch was normalized by the average power of the baseline data (−4 to −2 s before the task cue) (Song et al., 2018; Song and Kim, 2019). The mean and standard deviation for the peak amplitude of RP from the ROI and non-ROI channels in the three paradigms were compared by quantitative comparison. For statistical analysis, we performed a paired t-test on the peak values of ROI and non-ROI. For the non-ROI in the comparison, the amplitude of the negative peak for non-ROI candidate channels was averaged for the MI paradigm, and the non-ROI candidate with the largest peak amplitude was selected for the other EEG contamination paradigms.

After screening the frequency bands and channels, to extract the training data, raw EEG signals were resampled to 64 Hz and band-pass filtered using the determined frequency band in the ROI selection. The filtered signal was sliced to 3 s moving window, overlapping every 20 ms (50 Hz). We then applied a phase 1 classifier to each moving window. The data that fit the classifier and its lower peak existed between 0 and 2 s after the cue were selected as the training data. The average of the training data was used for the phase 2 classifier. During the MI-BCI session, online EEG signals were sliced to a 3 s moving window (50 Hz) and applied to the proposed classifier.

#### Performance evaluation

We evaluated the performance of the classifier during both the calibration and MI-BCI sessions. For the calibration session, we obtained offline simulation results of the classifier, and the actual online performance of the classifier was analyzed for the MI-BCI session. Based on the number of TPs and FPs, the performance was evaluated using sensitivity (Altman and Bland, 1994; Bhagat et al., 2016), selectivity (Altman and Bland, 1994; Chae et al., 2012), FP rate (Pfurtscheller et al., 2003; Leeb et al., 2007; Chae et al., 2012; Lew et al., 2012; Liu et al., 2013; Bhagat et al., 2016; Mrachacz-Kersting et al., 2017), and FP per minute (FPM) (Li et al., 2013; Rodriguez-Ugarte et al., 2017), as follows:


(3) $$\begin{array}{l} Sensitivity\;=\;\frac{Number\;of\;TP}{Number\;of\;trials}\times 100\;\left(\%\right) \end{array}$$

(4) $$\begin{array}{l} Selectivity\;=\;\frac{Number\;of\;TP}{Number\;of\;total\;detections}\times 100\;\left(\%\right) \end{array}$$

(5) $$\begin{array}{l} FP\;rate\;=\;\frac{Number\;of\;FP}{Number\;of\;trials}\times 100\;\left(\%\right) \end{array}$$

(6) $$\begin{array}{l} FP\;per\;minute\;=\;\frac{Number\;of\;FP}{Total\;elapsed\;rest\;period}\times 100\;\left(\%\right) \end{array}$$

For the quantitative comparison between offline and online results, we applied a paired t-test on the number of TPs and FPs from offline results of the MI paradigm in the calibration session and online results of the MI-BCI session. Note that as the number of participants in the healthy group was too small for statistical analysis, the analysis was applied in the case of a stroke group (*n* = 9) and in the case of all participants who participated in the MI-BCI session (*n* = 14).

To investigate the effect of the key idea in the proposed classifier for FP rejection, the classification without non-ROI channels was simulated using MI-BCI session data of healthy participants and stroke patient groups and compared with online classification results in the same MI-BCI session by calculating the rejection rate, as follows:


(7) $$\begin{array}{l} rejection\;rate\;=\;\frac{Number\;of\;rejected\;FP}{Number\;of\;FP\;without\;non\;ROI\;channels}\;\times 100\;\left(\%\right) \end{array}$$

## Results

### Calibration session

**Figure 6:**
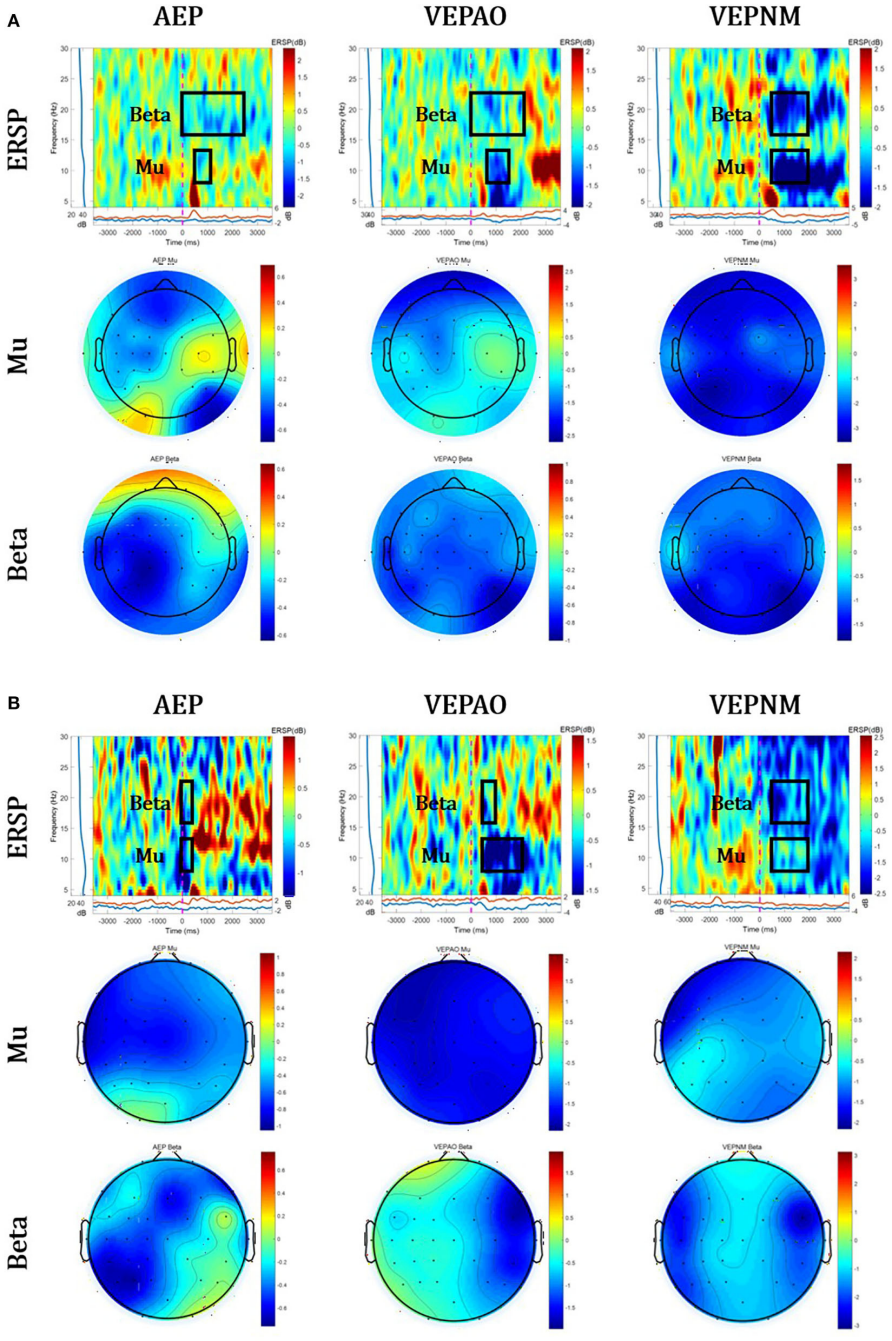
Group analysis of event-related spectral perturbation (ERSP) and its topographical map of Mu and Beta ERD. The red circles represent the average source area of EEG contaminations. **(A)** The healthy group and **(B)** stroke patient group (VEPAO: VEP with action observation; VEPNM: non–motor-related VEP).

Figure 6 shows the group analysis results for three paradigms (AEP, VEP with action observation, and non–motor-related VEP) of the calibration session to screen for EEG contamination.

**Figure 7:**
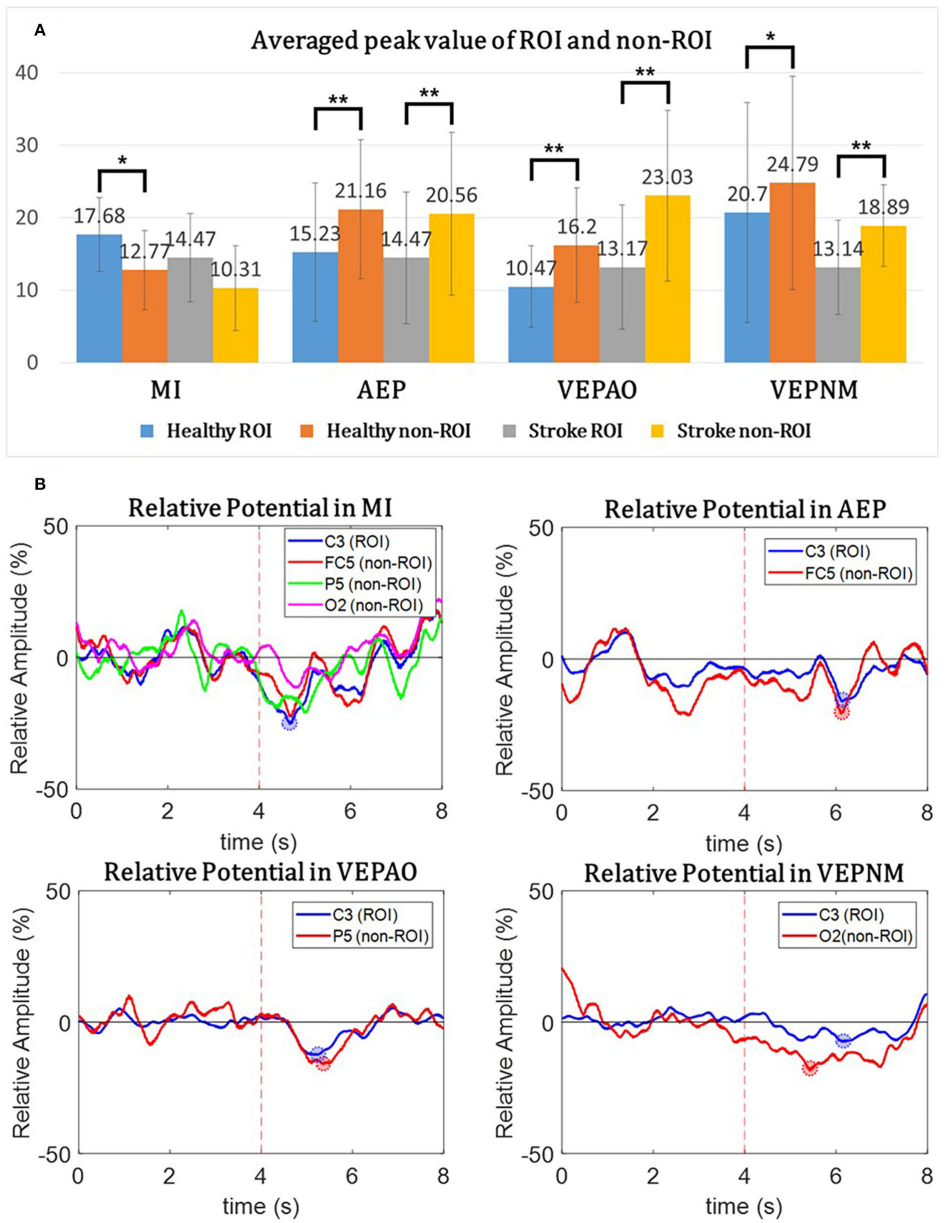
The relative potential from the region of interest (ROI) and non-ROI candidate channels, and the mean peak value of overall participants. **(A)** The average peak value of the ROI and non-ROI candidate channels for each participant group during each paradigm. The paired t-test is performed between the ROI and non-ROI candidate channels. Statistically mild significance (p<0.08) is described using a single asterisk (*) and statistical significance (*p* < 0.05) using a double asterisk (**). The candidates of the non-ROI channel for the MI paradigm are an average of three different non-ROI candidate channels. **(B)** The relative potential of the characteristic participant. The blue line represents the data of the ROI channels, and the red line represents the data from the non-ROI candidate channels. VEPAO, VEP with action observation; VEPNM, non–motor-related VEP.

Figure 7 shows the comparison results of the relative potential between the ROI and non-ROI candidate channels. Figure 7A illustrates the statistical analysis of the peak ERDs for each participant group during each paradigm. This result implies that the non-ROI candidate channels show stronger ERD signals when EEG contamination occurs due to visual/auditory stimulation. It should be noted that the largest peak amplitude of the non-ROI candidate channel exceeded the amplitude of the ROI channel for all paradigms except the MI paradigm in all trials and subjects, and the difference in the amplitude was statistically significant (p<0.05). As shown in Figure 7B, the relative potentials in the ROI channel tended to show larger ERD compared with that in the selected non-ROI candidate channels after the cue during the MI paradigm. The contamination paradigms tend vice versa.

**Figure 8:**
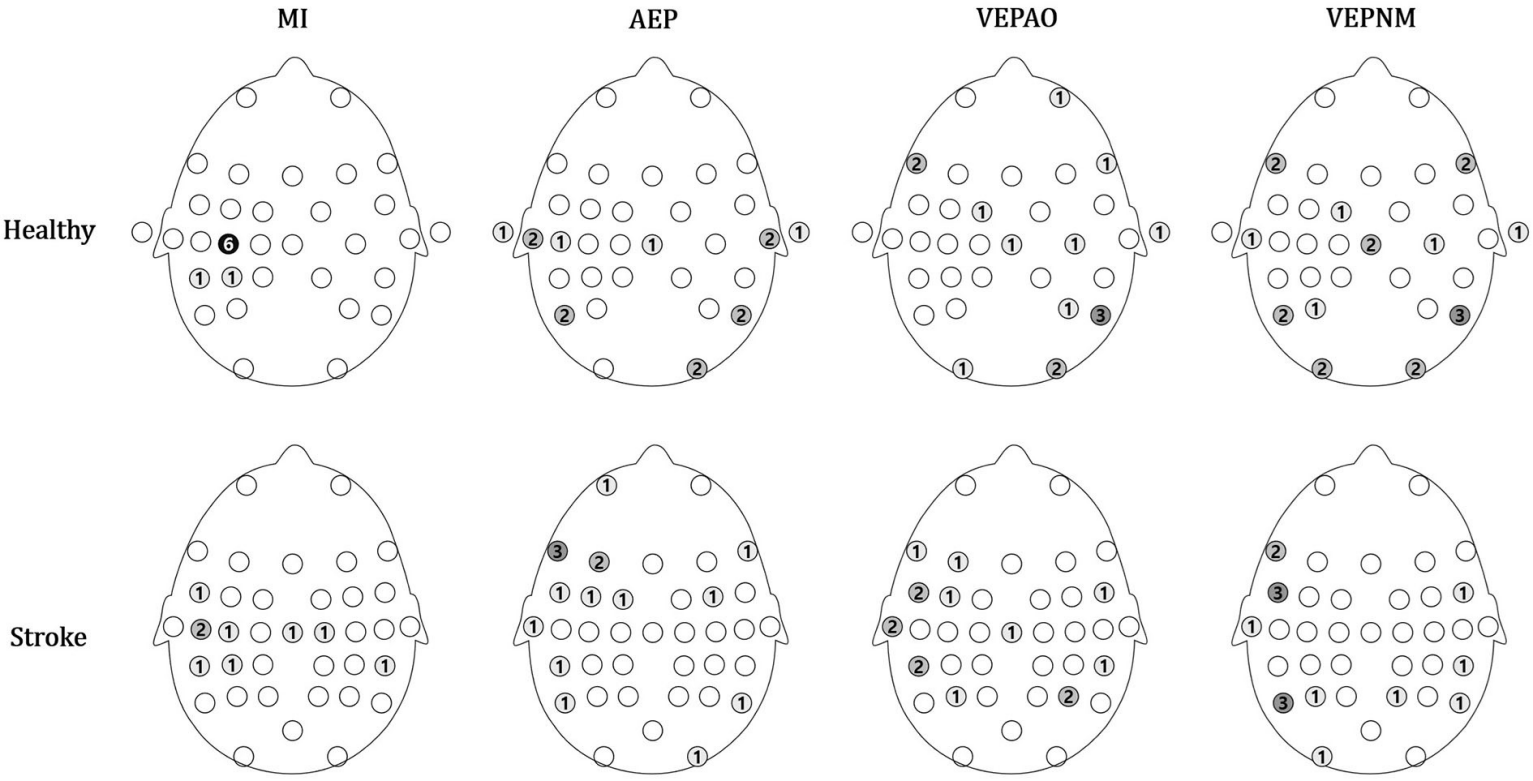
Group analysis of the topographical map for the number of regions of interest (ROI) and non-ROI candidate channels. The number of ROI channels is shown in blue, and the number of non-ROI candidate channels is shown in red. VEPAO, VEP with action observation; VEPNM, non–motor-related VEP.

The distributions of the chosen ROI and non-ROI candidate channels are illustrated as color maps in Figure 8. Here, the more the channels are concentrated, the darker the color. The ROI channels for the patient group were distributed in channels near the motor area (FC5, Cz, C3, C5, and CP5 for the right affected participants and C2 and CP6 for left affected participants), while most of the ROI channels were located on the motor cortex (C3 for six participants) and few were located in the somatosensory cortex (CP3 and CP5) in the healthy group (Figure 8). The candidates of the non-ROI channel for the healthy group were distributed on each diagonal end of the scalp, and those in the stroke group were mostly distributed in the left frontal (FT7 and FC5) and parietal lobes (P5).

### MI-BCI session

Tables 1, 2 describes the performance of the classifier in the offline simulation results and online MI-BCI sessions for all participants. The sensitivity was below 30% for both the healthy and stroke groups, and the FP rate was 12.67% in the healthy group and 8.52% in the stroke group. The non-ROI channels were widespread but mostly located in the temporal, parietal, and occipital lobes, as we targeted AEP and VEPs. For the patient group, the non-ROI channels were located similar to that of the healthy group; however, these channels were also located in the premotor cortex. Tables 2A,B describe the performance of the online MI-BCI session. For the healthy group, both sensitivity and selectivity increased compared with the offline simulation. For the patient group, the sensitivity increased; however, the selectivity slightly decreased.

**Table 1:** Performance of the classifier in offline simulation.

**Subject**	**Freq. band**	**ROI**	**Non-ROIs**	**TP**	**FP**	**FPR**	**FPM**	**Selec**.	**Sens**.
**(A) Offline simulation result of healthy group**									
S1	17–21 Hz	CP5	F7, Cz, P5	10	3	10	0.6	76.92	33.33
S2	20–25 Hz	C3	C4, P3, P6	13	8	26.67	1.6	61.90	43.33
S3	9–12 Hz	C3	F7, P3, P4	10	4	13.33	0.8	71.43	33.33
S4	17–22 Hz	CP3	Cz, T8, O1	8	3	10	0.6	72.73	26.67
S5	24–28 Hz	C3	T7, P6, O2	4	1	3.33	0.2	80	13.33
S6	10–14 Hz	C3	F7, T7, Cz	8	2	6.67	0.4	66.67	26.67
S7	18–22 Hz	C3	T10, P6, O2	4	0	0	0	100	13.33
S8	8–12 Hz	C3	F7, FC1, P5	4	2	6.67	0.4	66.67	13.33
Average	–	–	–	7.62	2.87	9.58	0.58	72.62	25.42
**(B) Offline simulation result of stroke group**									
P1	8–10 Hz	Cz	FC5, CP5, P5	8	5	16.67	1	61.54	26.67
P2	8–12 Hz	C5	P3, P4, F7	9	4	13.33	0.8	69.23	30
P3	16–22 Hz	FC5	F7, FC6, CP6	8	1	3.33	0.2	88.89	26.67
P4	10–12 Hz	C3	FC5, P5, O2	6	3	10	0.6	66.67	20
P5	8–10 Hz	Cz	F7, T7, P6	9	3	10	0.6	75	30
P6	19–22 Hz	C5	F7, F3, CP6	4	2	6.67	0.4	66.67	13.33
P7	8–12 Hz	CP6(L)	FC4, P5, O1	4	2	6.67	0.4	66.67	13.33
P8	14–17 Hz	CP5	F3, FC5, Cz	10	2	6.67	0.4	83.33	33.33
P9	17–23 Hz	C2 (L)	FC5, FC1, P5	4	1	3.33	0.2	80	13.33
Average	–	–	–	6.88	2.55	8.52	0.51	72.94	22.96

TP, true positives; FP, false positives; FPR, false positive ratio; FPM, false positive per minute.

**Table 2:** Performance online MI-BCI **(A)** in the healthy group and **(B)** in the stroke patients' group.

**Subject**	^*^Session interval (days)	**TP**	**FP**	**FPR**	**FPM**	**Selec**.	**Sens**.
**(A) Online result of healthy group**							
S1	40	10	4	13.33	1.33	71.43	33.33
S2	29	15	7	23.33	2.33	68.18	50
S3	22	10	3	10	1	76.92	33.33
S4	23	7	1	3.33	0.33	87.5	23.33
S5	18	10	0	0	0	100	33.33
Average	26.4 ± 8.6	10.4	3	10	1	77.61	34.67
**(B) Online result of stroke group**							
P1	14	14	6	20	1.5	70	46.67
P2	14	9	3	10	0.75	75	30
P3	14	10	3	10	0.75	76.92	33.33
P4	20	6	2	6.67	0.5	75	20
P5	14	8	3	10	0.75	72.72	26.67
P6	7	8	3	10	0.75	72.72	26.67
P7	8	12	7	23.3	1.75	63.16	40
P8	10	15	4	13.33	1	78.95	50
P9	5	12	6	20	1.5	66.67	40
Average	11.8 ± 4.7	10.44	4.11	13.70	1.03	71.76	34.81

TP, true positives; FP, false positives; FPR, false positive ratio; FPM, false positive per minute.

* Session interval indicates the interval between calibration session and online MI-BCI session.

**Figure 9:**
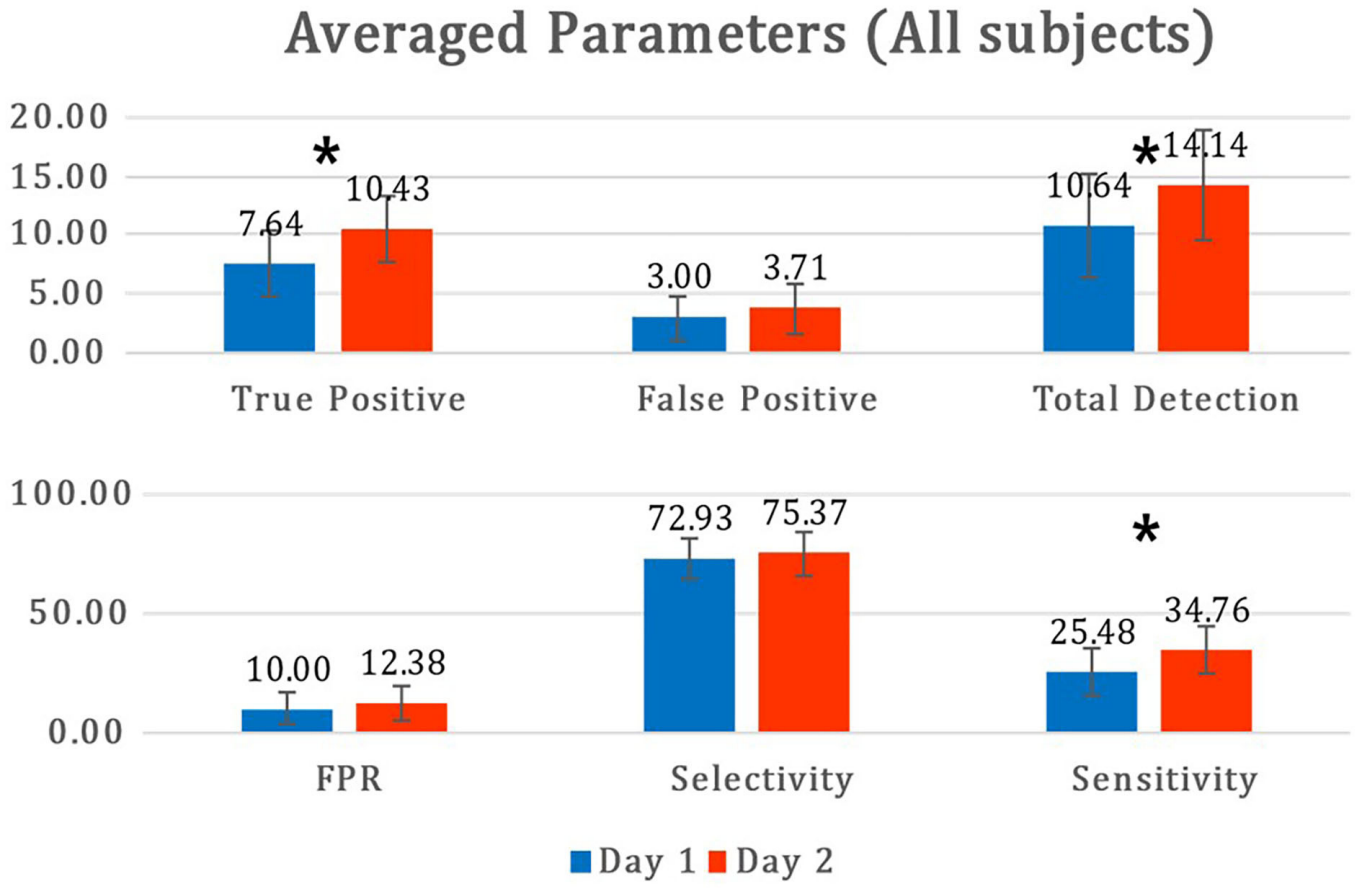
Mean and standard deviations of parameters between day 1 and day 2. The asterisk (*) is statistically significant (p<0.05) between days. FPR, false positive ratio.

Figure 9 describes the mean and standard deviation of parameters during the offline analysis of the MI paradigm (day 1) and online MI-BCI session (day 2). For the stroke group, the TPs showed a statistically significant increase (*p* = 0.015), and the FPs increased but were not significant (*p* = 0.071). The sensitivity also showed a significant increase as the sensitivity was dominantly related to the number of TPs. For all participants in the MI-BCI session (*n* = 14), the TPs showed a significant increase (*p* = 0.007), while the FPs did not (*p* = 0.246). The selectivity showed no significant difference between sessions for both groups (*p* = 0.792, *p* = 0.359 for the patient group and all participants' group each).

Table 3 describes the rejected number of FPs due to the non-ROI channel-based method for the healthy and stroke patient group. The most rejected FPs were 21, which is 75% of the FPs on P7. The highest and lowest rejection rates were 100 and 53.33%, respectively. The total rejection rate was 76.04% for all FPs.

**Table 3:** The number of false positives before and after applying the non-ROI channel-based method of the proposed classifier, and FP rejection in the online MI-BCI session.

**Subject**	**TP**	**FP (pre)**	**FP (post)**	**Rejected**	**Rejection rate (%)**
S1	10	13	4	9	69.23
S2	15	15	7	8	53.33
S3	10	10	3	7	70
S4	7	3	1	2	66.67
S5	10	2	0	2	100
P1	14	14	6	8	57.14
P2	9	20	3	17	85
P3	10	17	3	14	82.35
P4	6	18	2	16	88.89
P5	8	16	3	13	81.25
P6	8	18	3	15	83.33
P7	12	28	7	21	75
P8	15	20	4	16	80
P9	12	23	6	17	73.91
Average	146	217	52	165	76.04

TP, true positive; FP (pre), false positive before applying non-ROI channel-based method; FP (post), false positive after applying non-ROI channel-based method.

## Discussion

This study aimed to reduce FPs during rehabilitative MI-BCI that could result in wrong-directed brain plasticity. To this end, we proposed a classifier that contains single-channel-based MI detection and FP rejection using non-ROI channels.

As shown in the ERSP and band power of Figures 6, 7, EEG contamination elements (AEP, VEP with action observation, and non–motor-related VEP) affect the mu and beta bands in the motor area. The candidates of the non-ROI channel show larger amplitudes than the ROI channel when EEG contamination occurs due to visual or auditory stimulations. This means that the contamination elements originating from the non-ROI candidates can result in desynchronization at the ROI channel, and it could be detected as FP in the ROI. Despite the desynchronization at the ROI, there were significant band power differences between the ROI and non-ROI candidates (Figure 7). Therefore, it is feasible to find and reject contamination based on power differences.

Since the sources of the contamination elements on the scalp were differently distributed, it is essential to identify the sources through individual calibration (Figure 8). The MI signals of patients with stroke were also distributed around the motor area, which explains why finding a participant-specific ROI channel is an important task to improve MI-BCI performance for clinical application.

The experimental results suggest that our proposed MI-BCI system has a good FP rejection performance online, with a rejection rate of over 75%. As shown in Figure 9, both TP and FP tend to increase on day 2, compared with day 1. However, the mean selectivity did not show a significant difference and even increased slightly. This implies that our proposed algorithm is robust as it rejected a certain ratio of FP despite a significant session interval (day-to-day variation), even a month.

Our method consists of two phases of classifiers: (1) hand-crafted detection algorithm, and (2) correlation-based detection algorithm. The design intention was to use the phase 1 algorithm on reducing the number of sample windows and provides a synchronous-like state for assisting the phase 2 algorithm. To evaluate if the purpose was fulfilled, we performed an offline performance test of the phase 1 algorithm combined with non–ROI-based false positive rejection using day 1 MI data (Table 4). The results show relatively high sensitivity near 75% for both subject groups, with selectivity below but near 50%. These numbers indicate that the phase 1 algorithm combined with non-ROI technique provides a 50% chance of distinguishing true and false positives for the phase 2 algorithm with 25% of data loss.

**Table 4:** Performance of the phase 1 classifier in offline simulation.

**(A) Offline simulation result of healthy group**						
**Subject**	**TP**	**FP**	**FPR**	**FPM**	**Selec**.	**Sens**.
S1	23	20	0.67	4	0.53	0.77
S2	24	32	1.67	6.4	0.43	0.8
S3	21	18	0.6	3.6	0.54	0.7
S4	21	16	0.53	3.	0.57	0.7
S5	26	28	0.93	5.6	0.48	0.87
S6	24	27	0.9	5.4	0.47	0.8
S7	22	20	0.67	4	0.52	0.73
S8	22	24	0.8	4.8	0.48	0.73
Average	183	185	0.77	4.1	0.50	0.76
**(B) Offline simulation result of stroke group**						
P1	22	17	0.57	3.4	0.56	0.73
P2	23	31	1.03	6.2	0.43	0.77
P3	25	31	1.03	6.2	0.45	0.83
P4	19	22	0.73	4.4	0.46	0.63
P5	24	24	0.8	4.8	0.5	0.8
P6	26	25	0.83	5	0.51	0.87
P7	21	22	0.73	4.4	0.49	0.7
P8	21	23	0.77	4.6	0.48	0.7
P9	20	30	1	6	0.4	0.67
Average	201	225	0.83	5	0.47	0.74

TP, true positives; FP, false positives; FPR, false positive ratio; FPM, false positive per minute.

Several studies have applied BCI systems to patients with stroke (Hortal et al., 2015; Bhagat et al., 2016; Mrachacz-Kersting et al., 2017; Miladinović et al., 2020; Niazi et al., 2022). Table 5 compares the proposed method with existing studies. The main difference is that our system relies on a hand-crafted feature classifier, which is discriminated approach compared to spatial pattern-based machine-learning methods. Our method is originated from single-channel-based MI ERD detection, which cannot apply any spatial pattern-based machine-learning approach, but can only rely on the time-frequency aspect of the signal. Since our target signal has been clearly justified and it follows with the neurophysiological agreement throughout many studies (Pfurtscheller and Lopes Da Silva, 1999; Pfurtscheller et al., 2003; Kus et al., 2012; Nicolas-Alonso and Gomez-Gil, 2012; Sun et al., 2016; Jeong et al., 2019). We decided to use its nature to design features and algorithms without leaving them to machine learning; since machine-learning methods depend on the amount of training data, they are inappropriate to induce a decision rule like the proposed one, which consists of a large number of required features, out of such small datasets (patient's data) (Choi et al., 2018; Lee et al., 2021).

**Table 5:** Comparison of the method, experiment, and results with other studies applied to patients with stroke.

**Study**	**Task**	**Feature**	**Method**	**# obtained** **Calibration data/time spent**	**Session intervals**	**Subjects**	**# of channels used**	**Experiment paradigm**			**Performances**		
								**Task** **time (s)**	**Rest** **time (s)**	**T:R ratio/ total** **rest** **time per** **set (s)**	**Type** **of** **performance** **evaluation** **dataset**	**Sensitivity** **(%)**	**FPR** **(%)**
Hortal et al. (2015)	Motor Imagery, Grasping	ERD	SVM Spatial Pattern	304 task data 912 rest data/ 10 min.	1 day (no interval)	3 healthy 5 stroke	16	10	10	1:1/100	Online	H 82.9 S 45	H 19.2break S 15.0
Bhagat et al. (2016)	Motor Execution, Elbow	MRCP/EMG	SVM Spatial Pattern	160–320 data/53 min./day (not mentioned)	2-days of measurement 1-day calibration 2-days of the online trials (1 day interval)	4 stroke	60	15	5	1:0.33/100	Online	Day4 62.7 Day5 67.1	Day4 27.74 Day5 27.5
Mrachacz-Kersting et al. (2017)	Motor Execution, Reaching	MRCP	LPP-LDA	30 data/15 mins	1 day (no interval)	6 stroke	9	4	7	1:1.75/210	Online	1st 68.6 2nd 68.6	1st 33.6 2nd 21.2
Miladinović et al. (2020)	Motor Imagery, Grasping	ERD	(1) Source Power Co-Modulation^*^, (2) Spectrally Weighted CSP^**^, (3) Filter Bank CSP^***^	35–40 data per session (day), 15 sessions/ 10 mins	1 day (no interval)	5 stroke	15	5	2.1–2.8	1:0.48–0.56 /78.75–105	Offline	1) 83.0 2) 83.8 3) 85.1	1) 16.9break 2) 15.5 3) 15.5
Niazi et al. (2022)	Motor Execution, Ankle dor-siflexion	MRCP	Spatial pattern, likelihood	50 dataset, 6–10 min/(not mentioned)	2 day, ≥24 h	9 stroke	9	1.5	3–4	1:2–2.6/150	Online	82.68	15.25
Proposed	Motor imagery, Grasping	ERD	Shape, correlation	30 dataset/day 1:30 min. Day 2: 3 min.	2 days (avg. 17 days interval)	5 healthy 9 stroke	Day 1: 32 Day 2: 4	4	8	1:2/240	Online	H 35 S 34.8	H 10 S 13.70

* Meinel et al. (2019),

** Wei et al. (2008),

*** Park and Chung (2019).

An advantage of our method is the use of a small number of channels. It uses the smallest number (four) of channels in the MI-BCI session after a one-time calibration with 32 channels (Table 5). This can reduce the setting time of EEG for MI-BCI, which results in minimal fatigue for the patients and clinicians as well as better time efficiency of rehabilitative MI-BCI therapy. Since patients with stroke generally lose their attention and motivation easily, fatigue due to heavy EEG settings for the therapy would be critical for clinical application. All existing studies relied on spatial-based methods, such as LDA with spatial features (Blankertz et al., 2008; Lew et al., 2012) and LPP-LDA (Mrachacz-Kersting et al., 2017), Source Power Co-Modulation (Meinel et al., 2019), Spectrally Weighted Common Spatial Filter (CSP) (Wei et al., 2008), Filter Bank CSP (Park and Chung, 2019), and CSP with likelihood ratio method (Niazi et al., 2011, 2022); thus, these approaches suffer from heavy MI-BCI performance deterioration under a small number of channels (Arvaneh et al., 2011; Tam et al., 2011). Another advantage is the rare occurrence of FP during MI-BCI. For a fair comparison, we checked the false positives and experimental paradigms to calculate the FP rate (equation 5). The study by Hortal et al. (2015), Bhagat et al. (2016), Mrachacz-Kersting et al. (2017), and Miladinović et al. (2020) used the same calculation method as our study to report the FP rate. The study by Niazi et al. (2022) reported false positive per minute, true positive rate, and percentage of false positives over true positive. We inversely calculated the false positive rate using given parameters. Our system showed a 10% FP rate in the healthy group and 13.7% in the stroke group, which is the lowest FP rate compared with other existing studies (Table 5). It should be noted that our FP rates were obtained under the longest session interval between calibration and MI-BCI (Table 5). Along with the classifier used, the paradigm design also affected the occurrence of FP. Although a short task period is a disadvantage as patients with stroke generally require a longer time for MI due to chronic motor impairments, we used a shorter task period than other existing studies as extending the task period would cause misclassification between FP and TP. Moreover, the possibility of FP increases as the rest period becomes longer; however, our total rest period is the longest. Therefore, we believe that the FP rejection performance of the proposed system outperforms other existing studies, even though our paradigm design has disadvantages for FP.

The positive and negative effects of FP remain controversial (Levine et al., 2000; Barbero and Grosse-Wentrup, 2010; Alimardani et al., 2014). The exact effect of FP has not been determined; however, some studies claim that FP could be useful for improving MI in naïve BCI users (Alimardani et al., 2014). However, the goal of rehabilitative MI-BCI systems for patients with stroke is to guide them to perform correct MI based on neurophysiology to stimulate direct brain plasticity and improve the neuro-circuits. The most effective way to achieve this goal is by applying MI-BCI asynchronously; however, in this situation, the participant and/or clinician cannot notice whether the robotic feedback comes from TP or FP, without any cue. Since the nature of the training experience dictates the nature of neural plasticity (Kleim and Jones, 2008), if the patient is repeatedly exposed to the feedback induced by FP, it might lead to inappropriate brain plasticity. Therefore, reducing and minimizing FP would be essential for MI-BCI systems for neurorehabilitation.

Many patients with stroke who participated in the experiment commented that moving the rehabilitation robot due to TPs induced the feeling of body ownership like “I was controlling the robot hand” (Altman and Bland, 1994; Botvinick and Cohen, 1998; Michielsen et al., 2010; Evans and Blanke, 2013; Liang et al., 2016; Sun et al., 2016). In contrast, they also commented that the wrong robotic feedback due to FPs caused them to lose the agency and ownership of their hand, and this was frustrating and unpleasant. These comments show that rejecting FP is important for maintaining body ownership and agency in the MI-BCI system. However, some patients experienced anxiety and loss of interest when MI detection did not occur. This implies that low sensitivity could negatively affect MI-BCI therapy for some participants and work as an obstacle.

The proposed asynchronous MI-BCI system showed state-of-the-art FP rejection performance, while the sensitivity of the system was decreased compared with existing spatial-based approaches. The classifier design based on the characteristics of EEG contamination led our MI-BCI system to use a minimal number of channels for detecting MI ERD and for rejecting FP. Moreover, the classifier was insensitive to day-to-day variations. Therefore, we believe that the proposed system fits the conditions for practical use in clinics, fast setup time due to the small number of channels, and reliable performance owing to its insensitive day-to-day variation.

Our study mainly considered the EEG contamination on motor-related VEP, non–motor-related VEP, and AEP. However, our proposing algorithm can be applied to other EEG contaminations due to sensory stimuli, such as sound generated from medical devices and visual distractions in a rehabilitation facility, which show pseudo-MI ERD-like behavior. It is because the design of the algorithm intended to reject all pseudo-MI ERD originated from non-ROI channels which could be easily extended by screening the candidates of the non-ROI channel.

Despite many benefits, the limitation of our proposed system is its low sensitivity. The study of brain-switch on healthy subjects address that ERS-based single-channel MI detection could be achieved to a sensitivity of 59.2%, with FP rate below 10%, but sensitivity decreased to 28.4%, while ERD was used as a feature (Pfurtscheller and Solis-Escalante, 2008). This result suggests that ERD is a challenging feature compared to ERS. However, since our goal is to detect movement intention at the right timing to induce brain plasticity, ERS was inappropriate due to its delayed appearance. The averaged peak value during the MI paradigm in Figure 7A shows the low significance of ERD between ROI and non-ROI, which illustrates that some ERD from ROI channels might be rejected by the non–ROI-based classifier in some cases. This might imply that our non-ROI selection needs to be improved to consider the MI paradigm. Moreover, the second phase in the classifier, which was intended to detect samples with similar patterns to training data, might be too conservative because we only used 30 training data for each subject. In the viewpoint of inducing brain plasticity, whereas the FP-rejected asynchronous MI-BCI system induced cortical plasticity more than a typical self-paced asynchronous system with FP (Niazi et al., 2022), the correlation between sensitivity and cortical plasticity showed a negative association with significance (Jochumsen et al., 2019). The pieces of literature could illustrate that sensitivity does not significantly affect cortical activation compared to the FP rate. Nevertheless, the goal of the asynchronous MI-BCI system detects users' movement intention and gives them feedback at the proper time. Therefore, further research is needed to determine the appropriate level of sensitivity to encourage users, and it needs to be improved for better sensitivity in future. In future studies, we would like to evaluate the cortical activation difference between intensity-focused algorithms (high sensitivity, low FP) and specificity-focused algorithms (high FP) during MI-BCI training to verify the more important factor. Since the study did not evaluate the actual effect in the patients after the MI-BCI sessions, a long-term follow-up study would also become our next objective.

## Conclusion

This study aimed to develop the asynchronous MI-BCI system for neurorehabilitation use for people with stroke. To apply EEG-based BCI, we prioritized two factors: (1) small number of channels for user convenience and (2) reducing the number of FP to prevent wrong-directed brain plasticity and rehabilitation. We developed an MI ERD detection and FP rejection algorithm based on the time-frequency characteristics of MI ERD and EEG contaminations, with rippling characteristics of EEG signals. We categorized three EEG contaminations to assume as sources of FP: VEP during action observation, VEP during random images, and AEP with simple beep sound. These contaminations are easily found in the clinical rehabilitation environment, where our future system will be applied. We localized the surface source of each contamination and used a combination of those channels to reject FPs.

The designed algorithm was validated online for eight healthy subjects and nine patients with hemiplegic stroke. As a result, we showed the best FP rate compared to other asynchronous MI-BCI studies (10% for healthy subjects, 13.70% for patient subjects with stroke), while 76.04% of FP was rejected by applying a non-ROI channel method to single-channel detection-based algorithm. However, our system also showed the least sensitivity. The proposed system matched our intended objective; to reject FP conservatively. However, the sensitivity of the proposed system should be improved by further research.

## Data availability statement

The datasets presented in this article are prohibited due to privacy protection. Further inquiries can be directed to the corresponding author.

## Ethics statement

The studies involving human participants were reviewed and approved by Daegu Gyeongbuk Institute of Science and Technology. The patients/participants provided their written informed consent to participate in this study.

## Author contributions

JhK supervised the study, conceptualized designed the study, and acquired the funding and provided the resources for the study, and finalized the manuscript. MS and HJ implemented the proposed system and designed the experiments, acquired the data, and drafted the original manuscript. MS, HJ, S-HJ, and JbK recruited subjects and prepared IRB for the experiments. MS, HJ, and JbK processed, analyzed the data from the experiments, and interpreted the results from the data. All authors read and revised the manuscript and approved the final manuscript for publication.

## Funding

This research was supported in part by the National Research Foundation of Korea (NRF) and grant funded by the Korean government (MSIP) (No. 2022 R1 A2 C1008150).

## Conflict of interest

The authors declare that the research was conducted in the absence of any commercial or financial relationships that could be construed as a potential conflict of interest.

## Publisher's note

All claims expressed in this article are solely those of the authors and do not necessarily represent those of their affiliated organizations, or those of the publisher, the editors and the reviewers. Any product that may be evaluated in this article, or claim that may be made by its manufacturer, is not guaranteed or endorsed by the publisher.
